# Ambiguous species boundaries: Hybridization and morphological variation in two closely related *Rubus* species along altitudinal gradients

**DOI:** 10.1002/ece3.6473

**Published:** 2020-06-15

**Authors:** Makiko Mimura, Mihoko Suga

**Affiliations:** ^1^ Department of Biology Graduate School of Natural Science and Technology Okayama University Okayama Japan; ^2^ Graduate School of Agriculture Tamagawa University Tokyo Japan; ^3^ Present address: Sacred Heart School Tokyo Japan

**Keywords:** hybrid zone, introgression, morphology, species identification

## Abstract

Although hybridization frequently occurs among plant species, hybrid zones of divergent lineages formed at species boundaries are less common and may not be apparent in later generations of hybrids with more parental‐like phenotypes, as a consequence of backcrossing. To determine the effects of dispersal and selection on species boundaries, we compared clines in leaf traits and molecular hybrid index along two hybrid zones on Yakushima Island, Japan, in which a temperate (*Rubus palmatus*) and subtropical (*Rubus grayanus*) species of wild raspberry are found. Leaf sinus depth in the two hybrid zones had narrower clines at 600 m a.s.l. than the molecular hybrid index and common garden tests confirmed that some leaf traits, including leaf sinus depth that is a major trait used in species identification, are genetically divergent between these closely related species. The sharp transition in leaf phenotypic traits compared to molecular markers indicated divergent selection pressure on the hybrid zone structure. We suggest that species boundaries based on neutral molecular data may differ from those based on observed morphological traits.

## Graphical abstract

We compared clines of leaf morphology and molecular hybrid index at species boundaries of two closely related but ecologically distinct species. The leaf trait clines were steeper than molecular hybrid index clines. Species boundaries might be less obvious than one would conclude from the morphological traits.

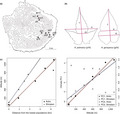

## 1 INTRODUCTION

Natural hybridization depends on the dispersal of genes between diverged lineages and is an evolutionary process that can lead to speciation and adaptive introgression. In plants, hybridization is a frequently occurring event, with evidence of natural hybridization or introgression recorded from 25% of plant species (Mallet, 2005); however, only a limited number (137) of natural hybrid zones have been observed (Abbott, 2017). Direct observation of hybrid zones offers a powerful means of observation of the mechanisms and conditions under which species boundaries are maintained (Barton & Hewitt, 1985; Brennan, Bridle, Wang, Hiscock, & Abbott, 2009; Gompert & Buerkle, 2016).

Species distribution ranges vary with time and space in response to climate change (Davis & Shaw, 2001), often resulting in the secondary contact and hybridization between lineages that are distinct but capable of interbreeding (Garroway et al., 2010; Mimura, Mishima, Lascoux, & Yahara, 2014). Consequences of hybridization following secondary contact may be varied, including extensive hybridization and species convergence (Hegde, Nason, Clegg, & Ellstrand, 2006; Ridley, Kim, & Ellstrand, 2008), adaptive introgression (Whitney, Randell, & Rieseberg, 2010), stable parapatric species boundary formation (Butlin, Ferris, Gosalvez, Hewitt, & Ritchie, 1992), extinction (Wolf et al., 2001), formation of new hybrid species (Mallet, 2007; Rieseberg, Carter, & Zona, 1990), and the replacement of one species by another (Dasmahapatra et al., 2002).

Interactions between gene dispersal (gene flow) and selection determine the formation and clines of hybrid zones, and a number of hybrid zone conceptual models have been constructed, such as the tension, bounded hybrid superiority, and mosaic hybrid zone models, to describe likely ecological processes that operate in these zones. Tension zone models assume that hybrids have low fitness relative to parental species (Barton & Hewitt, 1989). Intrinsic selection against hybrids can lead to narrow clines of neutral molecular markers (Martinsen, Whitham, Turek, & Keim, 2001). Bounded hybrid superiority zone models assume greater levels of fitness of hybrids than either parent species in intermediate habitats and extrinsic hybrid selection (Moore, 1977). Hybrid superiority can lead to the exclusive occupation by hybrids and smooth clines of the hybrid zone (Hamilton, Lexer, & Aitken, 2013). Mosaic hybrid zone models assume hybrid zones occur in heterogeneous environments in which parental species co‐occur (Harrison, 1986). However, the assignment of hybrid zones to the most appropriate model is complex; for example, a narrow tension zone was found to be evident for flower color and associated gene polymorphism in *Antirrhinum*, but not for other single nucleotide polymorphisms (Whibley et al., 2006).

Comparison of morphology and neutral genetic marker clines may illustrate a balance between selection and dispersal (Brennan et al., 2009; Gompert, Mandeville, & Buerkle, 2017). For example, steeper clines in phenotypic traits than molecular markers that may indicate divergent selection despite gene flow (Campbell, Faidiga, & Trujillo, 2018). The limited number of hybrid zones (Abbott, 2017) may reflect problematic identification of hybrid zones and hybrids, because only early generations of hybrids may be recognized based on phenotypic traits. Alternatively, plant hybrid zones may indeed be rare, as a consequence of reproductive isolation and lower fitness of hybrids (Baack, Melo, Rieseberg, & Ortiz‐Barrientos, 2015). Observation of transitions in traits and dispersal along hybrid zones can facilitate the identification of selection processes against hybrids and increase understanding of species boundary dynamics.

The closely related *Rubus palmatus* Thunb. and *R. grayanus* Maxim. (raspberry), which form a closely related group (Okada, Kikuchi, Hoshino, Kunitake, & Mimura, 2020) in the subgenus *Idaeobatus*, diverged approximately one million years ago (Mimura et al., 2014) and are predominantly distributed in temperate and subtropical climatic regions, respectively. However, effects of climate change have driven secondary contact between these species and there are areas in which hybrid zones have formed, such as on Yakushima Island, Japan (Mimura et al., 2014). Yakushima Island is small (504.9 km^2^), but mountainous (1,936 m a.s.l.), where *R. grayanus* is generally distributed in the lowlands, while *R. palmatus* tends to be distributed in uplands. Temporal flowering peaks of the hybrid zone shifts along the altitudes on the island, starting in February in the lowlands (*R. grayanus*) followed by the uplands (*R. palmatus*) in April. A previous study of *R. palmatus* and *R. grayanus* on the island reported gradual changes in the genetic structure of the hybrid zones, comprising later generations of hybrids (F_N_ hybrids) and individuals backcrossed to both parental species (Mimura et al., 2014).

The smooth clinal changes in population genetic structure found in the previous study (Mimura et al., 2014) may indicate there is little intrinsic selection on hybrids, so that formation of tension zones within the hybrid zones on Yakushima Island is unlikely. However, our preliminary observations of *Rubus* hybrid individuals inhabiting intermediate altitudes found that leaf morphology metrics, which are important in species identification, appeared to be more similar to *R. grayanus* than to *R. palmatus*, indicating a cline in the phenotypic trait may be different from, perhaps steeper than, the molecular cline. The phenotypic differences may be a result of plastic responses to local environmental conditions; therefore, identification of genetic effects on morphological traits may confirm species plastic responses or hybridization processes under selection pressures in a hybrid zone.

In this study, we aimed to determine effects of dispersal and selection on phenotypic and molecular trait variations in *R. grayanus* and *R. palmatus* along altitudinal gradients within their hybrid zone on Yakushima Island to elucidate species boundary processes. Specifically, we compared leaf phenotypic trait clines to the population hybrid index, which was measured using neutral genetic markers (Mimura et al., 2014), to test for selection effects on leaf traits that are used in species determination. We also evaluated trait differences among parental species and natural hybrids subjected to the same, controlled environmental pressure, using a common garden experiment.

## 2 MATERIALS AND METHODS

### 2.1 Study sites and *Rubus* populations

**Figure 1:**
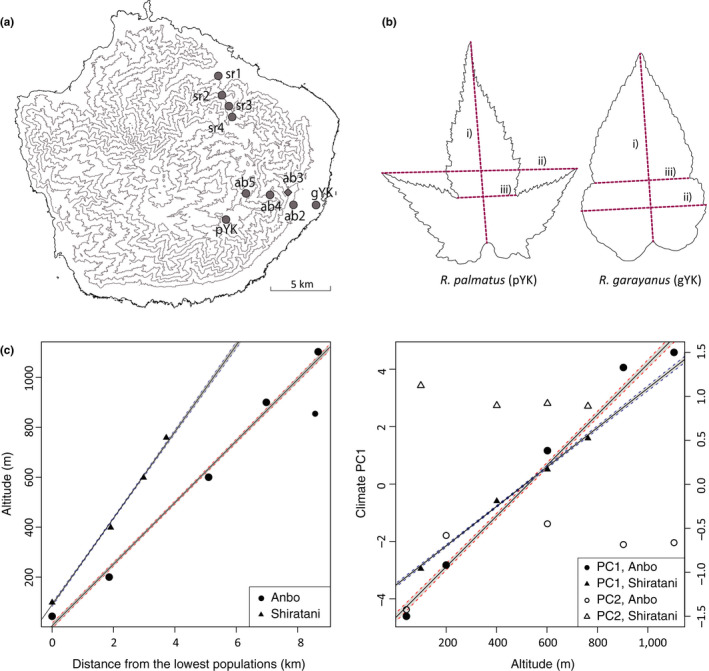
Location of transects at Anbo and Shiratani, parental leaf shape, and climate characteristics along altitudinal gradients. (a) location of study sites for leaf trait measurements (solid circles) and for hybrid index (solid circles + a solid diamond; Mimura et al., 2014); (b) leaf shapes of *R. palmatu*s and *R. grayanus* indicating measurement of sinus depth as shortest width/largest width (iii/ii) and leaf length:width ratio as leaf length divided by width (i/ii); (c) relationship between distance and altitude along the hybrid zone (left panel) and changes in climate PC1 and PC2 with altitude (right panel), where estimation of PCs was based on ten climate variables (Table 1); significant relationships (linear regressions) and 95% confidence intervals were plotted in red (Anbo) and blue (Shiratani)

We recorded leaf traits from nine *Rubus* populations (gYK, ab2, ab4, ab5, pYK, sr1, sr2, sr3, sr4) distributed along two transects located on trails at Anbo (ab, gYK, and pYK) and Shiratani (sr) on Yakushima Island, from where we had previously studied (Mimura et al., 2014; Table 1, Figure 1a). We also used the molecular hybrid index from the ten populations, including the population ab3, that we had previously estimated in Mimura et al., (2014). Distance between populations at the lowest (*R. grayanus*) and highest altitudes (*R. palmatus*) along the transects was estimated using Google Earth (Table 1; Anbo: 43–1,100 m a.s.l., 8.72 km; Shiratani: 100–760 m a.s.l., 3.72 km). Data for ten climate variables related to temperature and precipitation at the study sites were obtained from ClimateAP (Wang, Wang, Innes, Seely, & Chen, 2019; Table 1); the variables were highly correlated, so we estimated principal components (PC) in R (R Core Team, 2016) to generate independent climate PC variables that were analyzed using linear regression, with 95% confidence intervals for significant coefficients.

**Table 1:** Study site location and climate conditions. AHM, annual heat‐moisture index calculated as (MAT + 10)/(MAP/1,000); DD, degree‐days; MAT, mean annual temperature (°C); MWMT, mean warmest month temperature (°C); MCMT, mean coldest month temperature (°C); TD, temperature difference between MWMT and MCMT, or continentality (°C); MAP, mean annual precipitation (mm). Climate PC1 (90.6%) and PC2 (8.0%) are the first and second principal components, respectively, obtained based on these climate data.

Transect	Pop ID*^a^	Species	Hybrid Index*^b^	Altitude (m)	Distance (km)^b^	MAT	MWMT	MCMT	TD	MAP	AHM	DD < 0	DD > 5	DD < 18	DD > 18	Climate PC1	Climate PC2
Anbo	gYK	*R. grayanus*	0.00	43	0	19.0	26.9	11.0	15.8	3,392	8.5	0	5,069	788	1,175	−4.03	−1.48
	ab2		0.03	200	1.86	18.0	25.9	10.0	16.0	3,373	8.3	1	4,731	979	991	−2.43	−0.58
	ab3		0.16	400	3.08	16.9	24.9	8.8	16.1	3,365	8.0	1	4,343	1,180	802	−1.03	−0.07
	ab4		0.27	600	5.10	15.7	23.7	7.5	16.2	3,418	7.5	2	3,918	1,449	618	1.07	−0.45
	ab5		0.90	900	6.98	14.3	22.4	6.0	16.4	3,465	7.0	5	3,440	1,770	436	3.92	−0.50
	pYK	*R. palmatus*	1.00	1,100	8.72	14.2	22.2	5.8	16.3	3,467	7.0	5	3,391	1803	416	3.88	−0.76
Shiratani	sr1		0.00	100	0	17.9	25.8	9.7	16.1	3,272	8.5	1	4,685	1,007	971	−2.62	1.15
	sr2		0.15	400	1.91	16.6	24.6	8.4	16.2	3,314	8.0	2	4,228	1,266	754	−0.51	0.93
	sr3		0.45	600	2.98	16.0	24.0	7.7	16.3	3,335	7.8	2	4,018	1,391	663	0.48	0.98
	sr4		0.98	760	3.72	15.6	23.6	7.3	16.3	3,351	7.6	3	3,866	1,443	629	1.28	0.76

Note

^a^ Population IDs and molecular hybrid indices were obtained fromMimura et al. (2014)

^b^ For each trail, distances are from the population at the lowest altitude (gYK and sr1).

#### 2.1.1 Leaf traits

New leaves tend to be produced in *Rubus* species continuously through the growing season, so at the beginning of August 2017, we collected three fully developed leaves from current‐year branches of the nine study populations of *R. grayanus* and *R. palmatus* on the island (*n* = 151; average of 16.8 individuals per population). All leaves were scanned on the sampling day for measurement of leaf area, length and width of leaf blade, and sinus width (shortest width between sinuses of a blade) using ImageJ (Schneider, Rasband, & Eliceiri, 2012). The depth of sinus was calculated as width of sinus divided by width of blade, and leaf length:width ratio was calculated as leaf length divided by width (Figure 1b). Leaf dry mass per area (LMA) was estimated from six leaf disks (10 mm in diameter) from the leaves. The mean values of replicated leaves per plant were used for the following data analyses. In addition to morphological leaf traits, we also measured chlorophyll and carotenoid contents in fresh leaves, where three leaf disks were collected from the leaf samples and placed in 1.5 ml of 80% aqueous acetone at 4°C overnight to extract chloroplasts. Then, absorbance of the colorless leaf disks was measured at 470, 646.8, and 663.2 nm using a spectrophotometer (UV‐2450, Shimadzu Corp.), and concentrations of chlorophyll *a*, chlorophyll *b*, and carotenoids were estimated according to Wellburn (1994).

#### 2.1.2 Cline analysis and migration estimation

The leaf trait data and hybrid index were fitted to a series of one‐dimensional geographic cline models (Gay, Crochet, Bell, & Lenormand, 2008; Szymura & Barton, 1986, 1991) using the Metropolis–Hastings Markov Chain Monte Carlo algorithm, implemented in the R package “hzar” (Derryberry, Derryberry, Maley, & Brumfield, 2014). For leaf trait data, individual measurements were transformed to normal distribution, when needed, and scaled to values between zero and one for each transect, before clines were fitted to the population means (Derryberry et al., 2014) in five models with contrasting exponential tails (none fitted, left or right tail only, mirror tails, both tails) for best model selection. A null model estimation of no cline was calculated against distance and altitude.

For molecular clines, we used molecular hybrid index that had been previously estimated in Mimura et al. (2014). They calculated population means of maximum likelihood estimates of hybrid index (Buerkle, 2005) for the ten populations (16 samples each population) on Yakushima Island based on the partial sequences of seventeen nuclear functional genes, which were all statistically neutral. Calculation of the molecular hybrid index was fitted to the fifteen cline models and null model that represented possible combinations of three trait intervals (fixed to 0 and 1, observed values, estimated values) and the five different tails described above (Derryberry et al., 2014). Each model was run with chain length of 100,000 steps with 10,000 burn‐in steps, and the best models were selected based on corrected Akaike information criteria (AICc).

Rates and directions of gene dispersal among populations along the hybrid zones were estimated using the sequence data (Mimura et al., 2014), on which the hybrid index was based, using Bayesian inference in MIGRATE‐*N* (version 3.6.11; Beerli & Felsenstein, 2001). Although flowering time in the hybrid zones may overlap between neighboring populations, it shifts with altitude, so movement of pollinators across the hybrid zones was assumed to be limited to among in‐flower patches; thus, we assumed a stepping‐stone migration modeling approach along each transect to allow migration rates to vary between neighboring populations. The Bayesian inference was performed with one single long run of static heated chains (temperatures: 1.0, 1.5, 3.0, and 10,000) and, after discarding the first 25,000 trees, we sampled and recorded every 100th of 1,000,000 steps in each of the ten replicates. Then, posterior probabilities of theta and mutation scaled immigration rates (m/μ) along the transects were estimated.

### 2.2 Common garden experiments

We established a common garden experiment to standardize and control for environmental effects on phenotypic traits among the parental species and natural hybrids, to understand the role of genetic processes in trait variation. Twelve plant suckers from each parental populations (pYK and gYK) and natural hybrids were collected from the two hybrid zones at 600 m a.s.l. (8 individuals from ab4 and 4 individuals from sr3) in 2015 and grown in a common garden at Tamagawa University, Tokyo, Japan. The molecular data in the previous study confirmed that pYK and gYK populations at Anbo transect comprised “pure” individuals of *R. palmatus* and *R. grayanus*, respectively, while there were a few backcrossed individuals in the populations of both ends of Shiratani transect (Mimura et al., 2014). In 2016, the plants were transplanted into larger plastic pots (30 cm in diameter, 12.8 L), and in spring 2020, three fully developed fresh leaves per individual were collected and scanned on the sampling day for measurement of the same leaf traits using ImageJ (Schneider et al., 2012) and analysis of chlorophyll and carotenoid content, as described above (Section 2.1.1). A Wilcoxon rank sum test, with Bonferroni *p*‐value adjustment, was performed to compare the leaf traits among the groups (two parental species and hybrids). Analysis of Variance (ANOVA) was also conducted for each trait with the three groups as a fixed effect and the hybrid zones as a random effect. To test correlation among all traits, we used pairwise Spearman rank correlation analysis.

## 3 RESULTS

### 3.1 Transect climate and environmental characteristics

Transect distance increased linearly with altitude (Figure 1c, Table 1), where the transect at Shiratani was steeper than at Anbo. Climate variables tended to be correlated (average of pairwise correlations, |*r*| = 0.88) and contributed to climate PC1 that explained 90.6% of total variation in climate conditions (Table S1). Greater PC1 values indicate cooler and wetter conditions and were linearly related to altitude. Climate PC2 explained 8.0% of the total variance in climate conditions (lower mean annual precipitation and greater difference in temperature between the mean warmest and coldest months).

### 3.2 Leaf trait differentiation under controlled environmental conditions

**Figure 2:**
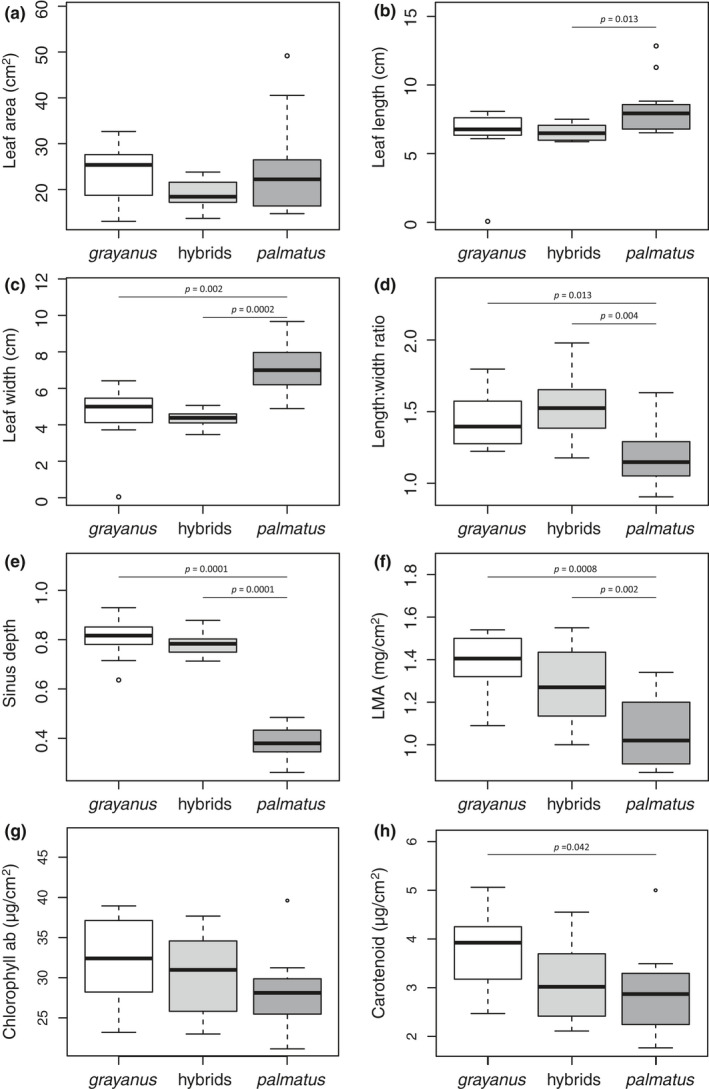
Boxplots of differences in leaf traits (Wilcoxon rank sum test, *p* < 0.05) of the parental populations and the natural hybrids at 600 m a.s.l. under controlled environmental conditions in the common garden experiment. LMA: leaf mass per area

There were differences in four phenotypic leaf traits between the parental species under controlled environmental conditions in the common garden experiment (Figure 2), where leaf width was greater (*p*‐value = 0.002; Figure 2c) and leaf length:width ratio, sinus depth, and LMA were lower in *R. palmatus* (*p*‐value = 0.013, 0.0001, 0.0008, respectively; Figure 2d, e, f). Thus, *R. palmatus* had lighter and more orbicular leaves with deeper lobes, whereas *R. grayanus* had thick, long, and shallow sinuses. Leaf content of carotenoid by leaf area was significantly lower in *R. palmatus* than in *R. grayanus* (*p*‐value = 0.042; Figure 2h). Leaf width and leaf length:width ratio, sinus depth, and dry weight of the natural hybrids differed from those in *R. palmatus*, but not *R. grayanus*. The content of chlorophyll and carotenoid of the natural hybrids did not differ from the parental populations and had intermediate values between parental species. Significant differences were also found in the comparison among the groups (two parental populations and hybrids) using ANOVA in leaf length (*F* = 3.80, *p*‐value = 0.033), leaf width (*F* = 16.56, *p*‐value < 0.001), leaf length:width ratio (*F* = 13.75, *p*‐value < 0.001), sinus depth (*F* = 153.5, *p*‐value < 0.001), LMA (*F* = 13.34, *p*‐value <0.001) and carotenoid (*F* = 3.38, *p*‐value = 0.047), but not in leaf area and chlorophyll ab.

### 3.3 Traits and hybrid index cline models

**Figure 3:**
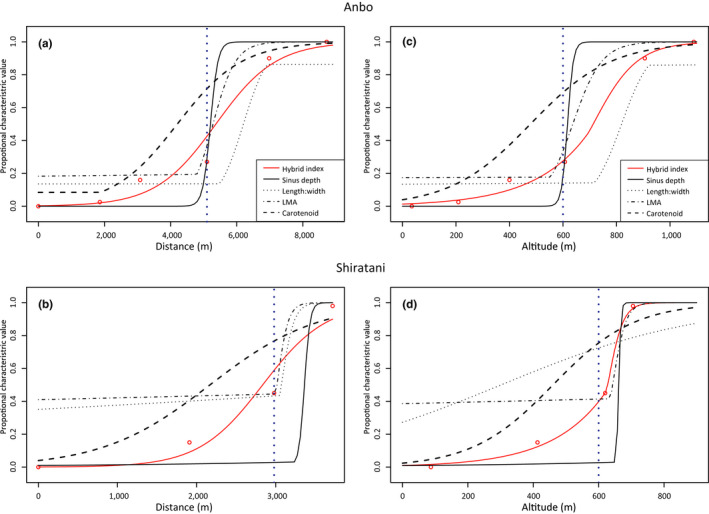
Maximum likelihood of best model estimates of clines in variance in the leaf traits and the molecular hybrid index with distance from populations at the lowest altitudes (*R. grayanus* populations) and with altitude along the Anbo (a, c, respectively) and Shiratani (b, d, respectively) *Rubus* spp. hybrid zones. Blue dashed lines indicate 600 m a.s.l.

We selected traits for the cline analysis from the common garden experiment that differed between the parental species (LMA, length:width ratio, sinus depth, and carotenoid content) and were noncorrelated (*r* < 0.70 between trait pairs); the population hybrid index and field transect data for these leaf traits were fitted to the clinal models by distance and altitude. Estimates of the best models for distance (Figure 3a, b) and altitude (Figure 3c, d) were similar for the two transects; the null models were not selected for the tested traits or the hybrid index. There was a gradual and smooth transition in the hybrid index along the transects (red lines in Figure 3), whereas there were clear transitions in leaf morphology (sinus depth, leaf Length:width, and LMA). Cline centers of the hybrid index were located at 5.38 and 2.83 km along the Anbo and Shiratani transects, similar to those of the sinus depth, dry weight, and Length:width ratio leaf traits (Table 2). However, cline widths for these leaf traits were narrower than the molecular hybrid index in both hybrid zones and cline centers of carotenoid content shifted toward *R. grayanus* (Table 2). Although transect length differed between Anbo and Shiratani, the centers of the transition of the three leaf traits were similar and occurred around 600 m a.s.l. (Figure 3c, d); at this altitude, the models estimated greater likelihood of phenotypes with *R. grayanus*‐like sinus depth.

**Table 2:** Parameters estimated by best‐fitting cline models for molecular hybrid index and leaf trait clines with distance from populations at the lowest altitudes (*R. grayanus* populations). The estimated cline center, measured as the distance from the population at the lowest altitude (*R. grayanus*), and cline width (1/maximum slope) were estimated in R using “hzar” (Derryberry et al., 2014); two log‐likelihood units of the parameter estimates are shown in parentheses.

Hybrid zone	Trait	Cline center (km)	Cline width (km)
Anbo	Hybrid index	5.38 (4.72–6.04)	3.63 (2.46–5.48)
	Dry weight (mg/cm^2^)	5.24 (4.93–6.57)	1.25 (0.25–3.97)
	Leaf length:width ratio	6.08 (5.47–7.11)	1.60 (0.07–4.77)
	Sinus depth	5.17 (5.10–5.97)	0.30 (0.02–3.24)
	Carotenoid (μg/cm^2^)	4.28 (3.51–4.97)	3.65 (1.71–5.60)
Shiratani	Hybrid index	2.83 (2.49–3.15)	1.61 (0.99–2.86)
	Dry weight (mg/cm^2^)	3.15 (2.81–3.68)	0.28 (0.05–1.88)
	Leaf length:width ratio	2.99 (2.74–3.56)	1.02 (0.43–2.41)
	Sinus depth	3.23 (3.01–3.68)	0.17 (0.03–0.70)
	Carotenoid (μg/cm^2^)	2.17 (1.68–2.64)	2.73 (1.72–4.03)

### 3.4 Estimation of migration rates between neighboring populations

**Figure 4:**
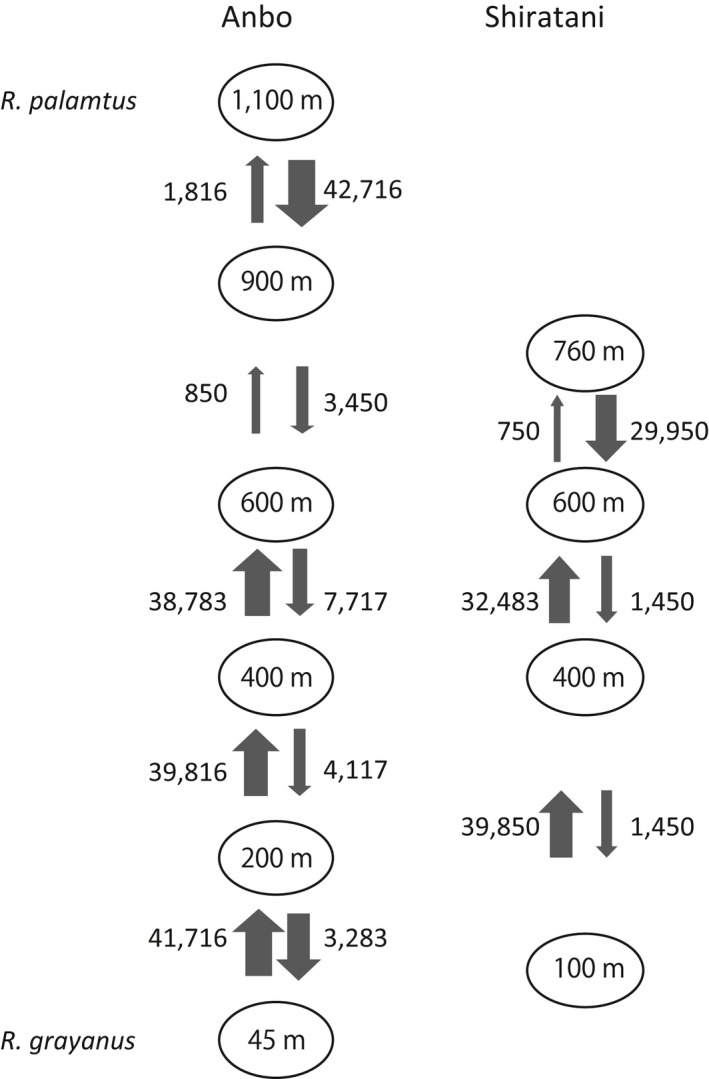
Estimated migration rates (m/μ), based on sequence data from Mimura et al. (2014), between neighboring populations at altitudes along the two hybrid zones. The model assumed free, stepping‐stone migration between neighboring populations

Based on the assumption of stepping‐stone migration, estimated migration rates along the hybrid zones were asymmetric at the altitudinal extremes, where migration rates between neighboring populations were greater from lower to higher altitudes at low altitudes and the reverse at higher altitudes (Figure 4). Thus, the populations at c. 600 m a.s.l. on the two transects were dominated by immigrants.

## 4 DISCUSSION

### 4.1 Hybrid zone structure and gene dispersal

The smooth clines in neutral molecular hybrid indices in the two hybrid zones in this study indicated a lack of strong dispersal barriers between the parental species. Survival and fitness of hybrids characterize hybrid zone structure; for example, lower fitness of post‐F_1_ generations was found to create a tension zone in *Senecio* (Brennan et al., 2009). In some cases of bounded hybrid superiority zones, there may be an absence of later generations of hybrids, but the presence of F_1_ hybrids, as has been reported for *Rhododendron* (Milne, Terzioglu, & Abbott, 2003) and *Utricularia* (Kameyama, Toyama, & Ohara, 2005), or higher levels of fitness of later generations of hybrids in habitats intermediate to parental species and a smooth molecular cline, such as in *Picea* (Hamilton et al., 2013). Previously, we found that the *Rubus* hybrid zones largely comprised backcrossed and later generations of hybrids, based on interspecific heterozygosity, indicating hybrid survival and reproduction (Mimura et al., 2014), and an artificial crossing experiment between the parental species produced viable seeds (Mimura et al. unpublished data). Although hybrid fitness was not directly evaluated, the smooth molecular cline indicates that strong intrinsic selection against hybrids is unlikely in the hybrid zones.

When we closely examined rates and direction of migration in the hybrid zones, we detected opposing directions in asymmetric gene flow between neighboring populations below and above 600 m a.s.l., where migration tended to flow from the extremes of the hybrid zones toward their centers. Asymmetric gene flow may be a result of adaptive introgression (Suarez‐Gonzalez, Hefer, Lexer, Cronk, & Douglas, 2018; Vonlanthen et al., 2012; Whitney et al., 2010), or a consequence of differences in population sizes or density that generate greater dispersal from larger populations than those of smaller populations (Lepais et al., 2009). The hybrid index we used in this study, which was based on statistically neutral molecular sequences (Mimura et al., 2014), should reflect neutral dispersal via seeds or pollen among neighboring populations. We observed no apparent declines in current population density at the centers of the hybrid zones, although overall, the population density of *R. palmatus* was lower than *R. grayanus*, perhaps due to increased patchiness in the distribution of *R. palmatus* at higher altitudes. Asymmetric dispersal indicates that hybrid population expansion and colonization from the upper and lower extremes of the hybrid zones, in which parental populations initially acted as source populations, were followed by frequent backcrossing toward spatially closer parental species.

### 4.2 Drastic changes in leaf traits at species boundaries

Phenotypic variation is driven by genetic and environmental pressure. Although the environmental conditions at the common garden experiment contrasted with those at the island study sites, they were standardized and controlled, to allow for analysis of genetic differences in *Rubus* phenotype and separation of phenotypic plasticity responses. There was genetic divergence between parental species leaf traits (Figure 2) and the cline analysis estimated that leaf sinus depth of the hybrids at 600 m a.s.l. on the two transects was more similar to *R. grayanus* than *R. palmatus* (Figure 3). These findings were supported by the common garden experiment that showed similar estimated clinal patterns in phenotypic differences in the leaf traits. Thus, modeling of our field data indicated that leaf traits, including those used for species identification, were mostly the product of genetic differences, rather than phenotypic plasticity. It is possible, however, that although the common garden experiment indicated contrasting photosynthetic strategies between parental species, differences among populations in the field may have been overestimated, because phenotypic plastic responses of chlorophyll content to different light conditions have been reported (Gratani, Covone, & Larcher, 2006).

The narrower leaf trait clines than molecular clines may be due to neutral diffusion or selection. Sharp phenotypic clines under neutral evolution may occur at relatively early stages of secondary contact, with limited migration rates between species (Gompert & Buerkle, 2016). The coalescent analysis estimated that hybrid zones in this study were formed following secondary contact more than c. 8,000 years ago, and later generations of hybrids have been observed (Mimura et al., 2014), indicating that the narrower phenotype than molecular clines did not emerge purely from neutral diffusion. The narrow tension zone of some leaf traits and smooth molecular clines in this study support the hypothesis that these traits were under extrinsic divergent or disruptive selection, despite the presence of gene flow. A sharp change in phenotypic traits compared to neutral markers has also been interpreted as a result of divergent or disruptive selection (Campbell et al., 2018; Scordato et al., 2017). It is possible that selection may act only on parts of genomic regions responsible for traits, while other regions may be unaffected in hybrid zones (Gompert et al., 2017; Whibley et al., 2006).

Altitudinal gradients may elicit strong environmental selection pressure in hybrid zones (Abbott & Brennan, 2014; Brennan et al., 2009), including from effects of variation in temperature, precipitation, and light availability (Körner, 2007). It is notable that the sharp transition from *R. grayanus*‐like to *R. palmatus*‐like phenotype leaf morphologies at c. 600 m a.s.l. was concordant between the Anbo and Shiratani hybrid zones, indicating that similar environmental factors may have acted on intermediate hybrids or phenotypes in these zones.

Leaf shape is associated with mean annual temperatures across continents, and plant communities under low temperature conditions tend to have higher proportions of species with toothed and lobed leaves than those under higher temperature conditions (Peppe et al., 2011). However, in this study, the selective pressures along the altitudinal gradient that may have sharpened the clines in leaf shape are unclear, because changes in the estimated climate along the hybrid zones were linear (Figure 3) and did not explain the sharp transition in leaf forms. Mechanisms that underpin the global trend in a correlation between leaf shape and temperature are yet to be clarified, but may be related to development of veins for support and water supply (Givnish & Kriebel, 2017), efficient photosynthesis in cool temperatures at the beginning of growing seasons (Baker‐Brosh & Peet, 1997; Royer & Wilf, 2006), and side‐effects as a consequence of bud‐packing (Edwards, Spriggs, Chatelet, & Donoghue, 2016). A recent study suggested the direct advantage of leaf teeth to carbon gain in early spring (Zohner, Ramm, & Renner, 2019). The changes in vegetation with altitude on Yakushima Island may affect light conditions that may act as a selective pressure in early spring. Further analyses are required to identify the influence of selective pressures on the clines in these hybrid zones, including measurement of environmental factors, such as spring light conditions and temperature at bud‐burst along an altitudinal gradient, and hybrid fitness, such as survival and photosynthetic efficiencies.

## 5 CONCLUSIONS

We observed genetic and morphological differences in leaf traits between two closely related, but ecologically distinct *Rubus* spp. in two hybrid zones on Yakushima Island, Japan. We repeatedly observed that the leaf traits, some of which indicated genetic differences between the two species, had much narrower cline widths than the molecular hybrid index. The results indicate extrinsic divergent selection pressure on *Rubus* leaf morphology at the hybrid zones, although it was difficult to identify an environmental factor as a selective pressure. Our results show that hybrid traits may quickly resemble those of their parents in the absence of strong reproductive barriers, and species boundaries may be less obvious than expected from the use of morphological traits in plant identification. This could explain why a low number of hybrid zones have been observed to date (Abbott, 2017), despite frequent hybridization in plants (Mallet, 2005). Hybridization and formation of hybrid zones increase with ongoing climate change and human activities, and our results indicate that genes penetrate closely related species at the margins of their ranges, where typical morphological differences can be retained. Hybridization may lead to increased risk of extinction (Todesco et al., 2016), but also increased chances of adaptive introgression, such as reported in a recent genomic study of evolutionary rescue in the context of water pollution (Oziolor et al., 2019). Thus, careful observation at species boundaries is necessary to identify and predict the outcomes of secondary contacts of species, especially under ongoing climate change conditions.

## CONFLICT OF INTERESTS

None declared.

## AUTHOR CONTRIBUTION

**Makiko Mimura:** Conceptualization (lead); Data curation (equal); Formal analysis (lead); Funding acquisition (lead); Methodology (equal); Supervision (lead); Writing‐original draft (equal); Writing‐review & editing (lead). **Mihoko Suga:** Data curation (equal); Methodology (equal); Writing‐original draft (equal).

## Supporting information

Table S1

## Data Availability

MIGRATE input and sequence files AND Morphological data: https://doi.org/10.5061/dryad.gb5mkkwmh

## References

[ece36473-bib-0001] Abbott, R. J. (2017). Plant speciation across environmental gradients and the occurrence and nature of hybrid zones. Journal of Systematics and Evolution, 55, 238–258. 10.1111/jse.12267

[ece36473-bib-0002] Abbott, R. J. , & Brennan, A. C. (2014). Altitudinal gradients, plant hybrid zones and evolutionary novelty. Philosophical Transactions Royal Soc B Biological Sci, 369, 20130346 10.1098/rstb.2013.0346 PMC4071520 24958920

[ece36473-bib-0003] Baack, E. , Melo, M. C. , Rieseberg, L. H. , & Ortiz‐Barrientos, D. (2015). The origins of reproductive isolation in plants. New Phytologist, 207, 968–984. 10.1111/nph.13424 25944305

[ece36473-bib-0004] Baker‐Brosh, K. F. , & Peet, R. K. (1997). The ecological significance of lobed and toothed leaves in temperate forest trees. Ecology, 78, 1250–1255. 10.1890/0012-9658(1997)078[1250:tesola]2.0.co;2

[ece36473-bib-0005] Barton, N. H. , & Hewitt, G. M. (1985). Analysis of hybrid zones. Annual Review of Ecology and Systematics, 16, 113–148. 10.1146/annurev.es.16.110185.000553

[ece36473-bib-0006] Barton, N. H. , & Hewitt, G. M. (1989). Adaptation, speciation and hybrid zones. Nature, 341, 497–503. 10.1038/341497a0 2677747

[ece36473-bib-0007] Beerli, P. , & Felsenstein, J. (2001). Maximum likelihood estimation of a migration matrix and effective population sizes in n subpopulations by using a coalescent approach. Proceedings of the National Academy of Sciences, 98, 4563–4568. 10.1073/pnas.081068098 PMC31874 11287657

[ece36473-bib-0008] Brennan, A. C. , Bridle, J. R. , Wang, A. , Hiscock, S. J. , & Abbott, R. J. (2009). Adaptation and selection in the *Senecio* (Asteraceae) hybrid zone on Mount Etna, Sicily. New Phytologist, 183, 702–717. 10.1111/j.1469-8137.2009.02944.x 19594693

[ece36473-bib-0009] Buerkle, C. A. (2005). Maximum likelihood estimation of a hybrid index based on molecular markers. Molecular Ecology Notes, 5, 684–687. 10.1111/j.1471-8286.2005.01011.x

[ece36473-bib-0010] Butlin, R. K. , Ferris, C. , Gosalvez, J. , Hewitt, G. M. , & Ritchie, M. G. (1992). Broad‐scale mapping of a hybrid zone between subspecies of *Chorthippus parallelus* (Orthoptera: Acrididae). Ecological Entomology, 17, 359–362. 10.1111/j.1365-2311.1992.tb01070.x

[ece36473-bib-0011] Campbell, D. R. , Faidiga, A. , & Trujillo, G. (2018). Clines in traits compared over two decades in a plant hybrid zone. Ann Bot‐london, 122, 315–324. 10.1093/aob/mcy072 PMC6070099 29800076

[ece36473-bib-0012] Dasmahapatra, K. K. , Blum, M. J. , Aiello, A. , Hackwell, S. , Davies, N. , Bermingham, E. P. , & Mallet, J. (2002). Inferences from a rapidly moving hybrid zone. Evolution, 56, 741–753. 10.1554/0014-3820(2002)056[0741:ifarmh]2.0.co;2 12038532

[ece36473-bib-0013] Davis, M. B. , & Shaw, R. G. (2001). Range shifts and adaptive responses to Quaternary climate change. Science, 292, 673–679. 10.1126/science.292.5517.673 11326089

[ece36473-bib-0014] Derryberry, E. P. , Derryberry, G. E. , Maley, J. M. , & Brumfield, R. T. (2014). hzar: Hybrid zone analysis using an R software package. Molecular Ecology Resources, 14, 652–663. 10.1111/1755-0998.12209 24373504

[ece36473-bib-0015] Edwards, E. J. , Spriggs, E. L. , Chatelet, D. S. , & Donoghue, M. J. (2016). Unpacking a century‐old mystery: Winter buds and the latitudinal gradient in leaf form. American Journal of Botany, 103, 975–978. 10.3732/ajb.1600129 27221280

[ece36473-bib-0016] Garroway, C. J. , Bowman, J. , Cascaden, T. J. , Holloway, G. L. , Mahan, C. G. , Malcolm, J. R. , … Wilson, P. J. (2010). Climate change induced hybridization in flying squirrels. Global Change Biol, 16, 113–121. 10.1111/j.1365-2486.2009.01948.x

[ece36473-bib-0017] Gay, L. , Crochet, P. , Bell, D. , & Lenormand, T. (2008). Comparing clines on molecular and phenotypic traits in hybrid zones: A window on tension zone models. Evolution, 62, 2789–2806. 10.1111/j.1558-5646.2008.00491.x 18752618

[ece36473-bib-0018] Givnish, T. J. , & Kriebel, R. (2017). Causes of ecological gradients in leaf margin entirety: Evaluating the roles of biomechanics, hydraulics, vein geometry, and bud packing. American Journal of Botany, 104, 354–366. 10.3732/ajb.1600287 28232316

[ece36473-bib-0019] Gompert, Z. , & Buerkle, C. A. (2016). What, if anything, are hybrids: Enduring truths and challenges associated with population structure and gene flow. Evolutionary Applications, 9, 909–923. 10.1111/eva.12380 27468308 PMC4947152

[ece36473-bib-0020] Gompert, Z. , Mandeville, E. G. , & Buerkle, C. A. (2017). Analysis of population genomic data from hybrid zones. Annual Review of Ecology Evolution and Systematics, 48, 207–229. 10.1146/annurev-ecolsys-110316-022652

[ece36473-bib-0021] Gratani, L. , Covone, F. , & Larcher, W. (2006). Leaf plasticity in response to light of three evergreen species of the Mediterranean maquis. Trees, 20, 549–558. 10.1007/s00468-006-0070-6

[ece36473-bib-0022] Hamilton, J. A. , Lexer, C. , & Aitken, S. N. (2013). Genomic and phenotypic architecture of a spruce hybrid zone (*Picea sitchensis* × *P. glauca*). Molecular Ecology, 22, 827–841. 10.1111/mec.12007 22967172

[ece36473-bib-0023] Harrison, R. (1986). Pattern and process in a narrow hybrid zone. Heredity, 56(3), 337–349. 10.1038/hdy.1986.55

[ece36473-bib-0024] Hegde, S. G. , Nason, J. D. , Clegg, J. M. , & Ellstrand, N. C. (2006). The evolution of California’s wild radish has resulted in the extinction of its progenitors. Evolution, 60, 1187–1197. 10.1554/05-634.1 16892969

[ece36473-bib-0025] Kameyama, Y. , Toyama, M. , & Ohara, M. (2005). Hybrid origins and F1 dominance in the free‐floating, sterile bladderwort, *Utricularia australis* f. *australis* (Lentibulariaceae). American Journal of Botany, 92, 469–476. 10.3732/ajb.92.3.469 21652424

[ece36473-bib-0053] Körner, C. (2007). The use of ‘altitude’ in ecological research. Trends in Ecology & Evolution, 22, 569–574. 10.1016/j.tree.2007.09.006 17988759

[ece36473-bib-0026] Lepais, O. , Petit, R. J. , Guichoux, E. , Lavabre, J. E. , Alberto, F. , Kremer, A. , & Gerber, S. (2009). Species relative abundance and direction of introgression in oaks. Molecular Ecology, 18, 2228–2242. 10.1111/j.1365-294X.2009.04137.x 19302359

[ece36473-bib-0027] Mallet, J. (2005). Hybridization as an invasion of the genome. Trends in Ecology & Evolution, 20, 229–237. 10.1016/j.tree.2005.02.010 16701374

[ece36473-bib-0028] Mallet, J. (2007). Hybrid speciation. Nature, 446, 279–283. 10.1038/nature05706 17361174

[ece36473-bib-0029] Martinsen, G. D. , Whitham, T. G. , Turek, R. J. , & Keim, P. (2001). Hybrid populations selectively filter gene introgression between species. Evolution, 55, 1325–1335. 10.1554/0014-3820(2001)055[1325:hpsfgi]2.0.co;2 11525457

[ece36473-bib-0030] Milne, R. I. , Terzioglu, S. , & Abbott, R. J. (2003). A hybrid zone dominated by fertile F1s: Maintenance of species barriers in Rhododendron. Molecular Ecology, 12, 2719–2729. 10.1046/j.1365-294x.2003.01942.x 12969475

[ece36473-bib-0031] Mimura, M. , Mishima, M. , Lascoux, M. , & Yahara, T. (2014). Range shift and introgression of the rear and leading populations in two ecologically distinct *Rubus* species. BMC Evolutionary Biology, 2014, 209 10.1186/s12862-014-0209-9 25344198 PMC4221717

[ece36473-bib-0032] Moore, W. S. (1977). An evaluation of narrow hybrid zones in vertebrates. Q Rev Biology, 52, 263–277. 10.1086/409995

[ece36473-bib-0033] Okada, A. , Kikuchi, S. , Hoshino, Y. , Kunitake, H. , & Mimura, M. (2020). Phylogeny and trait variation of Japanese *Rubus* subgenus *Ideaobatus* . Scientia Horticulturae, 264, 109150 10.1016/j.scienta.2019.109150

[ece36473-bib-0034] Oziolor, E. M. , Reid, N. M. , Yair, S. , Lee, K. M. , VerPloeg, S. G. , Bruns, P. C. , … Matson, C. W. (2019). Adaptive introgression enables evolutionary rescue from extreme environmental pollution. Science, 364, 455–457. 10.1126/science.aav4155 31048485

[ece36473-bib-0035] Peppe, D. J. , Royer, D. L. , Cariglino, B. , Oliver, S. Y. , Newman, S. , Leight, E. , … Wright, I. J. (2011). Sensitivity of leaf size and shape to climate: Global patterns and paleoclimatic applications. New Phytologist, 190, 724–739. 10.1111/j.1469-8137.2010.03615.x 21294735

[ece36473-bib-0036] R Core Team . (2016). R: A language and environment for statistical computing. Vienna, Austria: R Foundation for Statistical Computing https://www.R‐project.org/

[ece36473-bib-0037] Ridley, C. E. , Kim, S. , & Ellstrand, N. C. (2008). Bidirectional history of hybridization in California wild radish, *Raphanus sativus* (Brassicaceae), as revealed by chloroplast DNA. American Journal of Botany, 95, 1437–1442. 10.3732/ajb.0800119 21628151

[ece36473-bib-0038] Rieseberg, L. H. , Carter, R. , & Zona, S. (1990). Molecular tests of the hypothesized hybrid origin of two diploid *Helianthus* species (Asteraceae). Evolution, 44, 1498–1511. 10.2307/2409332 28564296

[ece36473-bib-0039] Royer, D. L. , & Wilf, P. (2006). Why do toothed leaves correlate with cold climates? Gas exchange at leaf margins provides new insights into a classic paleotemperature proxy. International Journal of Plant Sciences, 167, 11–18. 10.1086/497995

[ece36473-bib-0040] Schneider, C. A. , Rasband, W. S. , & Eliceiri, K. W. (2012). NIH Image to ImageJ: 25 years of image analysis. Nature Methods, 9, 671–675. 10.1038/nmeth.2089 22930834 PMC5554542

[ece36473-bib-0041] Scordato, E. S. C. , Wilkins, M. R. , Semenov, G. , Rubtsov, A. S. , Kane, N. C. , & Safran, R. J. (2017). Genomic variation across two barn swallow hybrid zones reveals traits associated with divergence in sympatry and allopatry. Molecular Ecology, 26, 5676–5691. 10.1111/mec.14276 28777875

[ece36473-bib-0042] Suarez‐Gonzalez, A. , Hefer, C. A. , Lexer, C. , Cronk, Q. C. B. , & Douglas, C. J. (2018). Scale and direction of adaptive introgression between black cottonwood (*Populus trichocarpa*) and balsam poplar (*P. balsamifera*). Molecular Ecology, 27, 1667–1680. 10.1111/mec.14561 29575353

[ece36473-bib-0043] Szymura, J. , & Barton, N. (1986). Genetic analysis of a hybrid zone between the fire‐bellied toads, *Bombina bombina* and *B. variegata*, near cracow in Southern Poland. Evolution, 40, 1141–1159. 10.2307/2408943 28563502

[ece36473-bib-0044] Szymura, J. , & Barton, N. (1991). The genetic structure of the hybrid zone between the fire‐bellied toads *Bombina bombina* and *B. variegata*: Comparisons between transects and between loci. Evolution, 45, 237 10.2307/2409660 28567861

[ece36473-bib-0045] Todesco, M. , Pascual, M. , Owens, G. , Ostevik, K. , Moyers, B. , Hübner, S. , … Rieseberg, L. (2016). Hybridization and extinction. Evolutionary Applications, 9, 892–908. 10.1111/eva.12367 27468307 PMC4947151

[ece36473-bib-0046] Vonlanthen, P. , Bittner, D. , Hudson, A. G. , Young, K. A. , Müller, R. , Lundsgaard‐Hansen, B. , … Seehausen, O. (2012). Eutrophication causes speciation reversal in whitefish adaptive radiations. Nature, 482, 357–362. 10.1038/nature10824 22337055

[ece36473-bib-0047] Wang, T. , Wang, G. , Innes, J. L. , Seely, B. , & Chen, B. (2019). ClimateAP: An application for dynamic local downscaling of historical and future climate data in Asia Pacific. Frontiers Agric Sci Eng, 4, 448 10.15302/j-fase-2017172

[ece36473-bib-0051] Wellburn, A. (1994). The spectral determination of chlorophylls a and b, as well as total carotenoids, using various solvents with spectrophotometers of different resolution. Journal of Plant Physiology, 144, 307–313. 10.1016/s0176-1617(11)81192-2

[ece36473-bib-0048] Whibley, A. C. , Langlade, N. B. , Andalo, C. , Hanna, A. I. , Bangham, A. , Thébaud, C. , & Coen, E. (2006). Evolutionary paths underlying flower color variation in *Antirrhinum* . Science, 313, 963–966. 10.1126/science.1129161 16917061

[ece36473-bib-0049] Whitney, K. D. , Randell, R. A. , & Rieseberg, L. H. (2010). Adaptive introgression of abiotic tolerance traits in the sunflower *Helianthus annuus* . New Phytologist, 187, 230–239. 10.1111/j.1469-8137.2010.03234.x 20345635

[ece36473-bib-0052] Wolf, D. E , Takebayashi, N. , & Rieseberg, L. H. (2001). Predicting the risk of extinction through hybridization. Conservation Biology, 15, 1039–1053. 10.1046/j.1523-1739.2001.0150041039.x

[ece36473-bib-0050] Zohner, C. M. , Ramm, E. , & Renner, S. S. (2019). Examining the support–supply and bud‐packing hypotheses for the increase in toothed leaf margins in northern deciduous floras. American Journal of Botany, 106, 1404–1411. 10.1002/ajb2.1379 31670844

