# Supplementary material for: Ambiguous species boundaries: Hybridization and morphological variation in two closely related Rubus species along altitudinal gradients
Source: Ecol Evol. 2020 Jun 15;10(14):7476–86. doi: 10.1002/ece3.6473 (PMC7391560; doi:10.1002/ece3.6473)
Supplement: Supplementary file 1 — Table S1 [file ECE3-10-7476-s001.docx]

Table S1 Summary of principal component analysis for climate data.

|  | PC1 | PC2 | PC3 | PC4 |
| --- | --- | --- | --- | --- |
| MAT | -0.331 | -0.066 | 0.124 | -0.137 |
| MWMT | -0.331 | -0.052 | 0.086 | -0.207 |
| MCMT | -0.331 | -0.099 | 0.105 | -0.121 |
| TD | 0.307 | 0.408 | 0.084 | -0.849 |
| MAP | 0.208 | -0.868 | -0.107 | -0.322 |
| AHM | -0.327 | 0.178 | 0.186 | 0.075 |
| DD<0 | 0.314 | -0.107 | 0.915 | 0.151 |
| DD>5 | -0.331 | -0.07 | 0.128 | -0.144 |
| DD<18 | 0.332 | 0.04 | -0.012 | 0.118 |
| DD>18 | -0.329 | -0.121 | 0.243 | -0.194 |
|  |  |  |  |  |
| Standard deviation | 3.011 | 0.896 | 0.337 | 0.131 |
| Proportion of Variance | 0.906 | 0.080 | 0.011 | 0.002 |
| Cumulative Proportion | 0.906 | 0.987 | 0.998 | 1.000 |
